# Genitofemoral Nerve Variation: An Attempt to Explain the Embryological Basis via a Case Report

**DOI:** 10.7759/cureus.61763

**Published:** 2024-06-05

**Authors:** Sanjukta Sahoo, KC Pradheep Kumar, Ravi K Narayan

**Affiliations:** 1 Anatomy, All India Institute of Medical Sciences, Bhubaneswar, IND

**Keywords:** nerve block, neuralgia, anatomic variation, branching, genitofemoral nerve

## Abstract

The genitofemoral nerve (GFN) presents with a variable course in nearly half of the population. This variation can be seen in its availability, course, and branching. Here, a notable case during a cadaveric dissection revealed an unusually high bifurcation of the GFN on the left side, contrasting with the typical bifurcation observed on the right. This divergence was highlighted using colored markers to aid educational visualization, facilitating a comprehensive learning experience about the nerve's variability and its functional implications, such as the cremasteric reflex. Embryologically, these variations stem from the migratory paths of myotomes during development, influenced by extrinsic signals and growth factors. Despite the high incidence of anatomical variability, the muscular structure remains consistent, suggesting that the nerve's formation is more susceptible to developmental shifts than the muscles it innervates. Clinically, understanding GFN variations is crucial due to the nerve's involvement in conditions like genitofemoral neuropathy, which can arise from surgical procedures. Accurate knowledge of these variations aids in precise diagnostic and therapeutic interventions, reducing complications, and enhancing patient outcomes in lower abdominal and groin surgeries. However, further research is needed to elucidate the exact embryological and genetic underpinnings of these variations.

## Introduction

The genitofemoral nerve (GFN) is a mixed nerve that arises from the lumbar plexus, especially the anterior rami of spinal nerves L1 and L2. It originates in the substance of the psoas major muscle, continues inside, pierces the muscle to lie on its anterior surface, and courses towards the inguinal ligament retroperitoneally. Along its course, the nerve is in posterior relation to the ureter, gonadal vessels, and abdominal vessels (the left colic artery and the inferior mesenteric vein on the left side; the ileocolic artery and vein on the right side). In typical anatomy, the nerve divides into two terminal branches, femoral (lumboinguinal) and genital, after piercing the psoas fascia just above the inguinal or Poupart’s ligament. Further, the femoral branch travels beneath the inguinal ligament to enter the femoral canal and acts as a cutaneous nerve for the upper anterior aspect of the thigh after piercing the anterior lamina of the femoral sheath. It also passes through the cribriform fascia of the saphenous opening of the fascia lata of the thigh to supply the upper, anterior, and medial sides of the thigh. The genital branch, also known as the external spermatic nerve, enters the inguinal canal through the deep inguinal ring. In men, it follows the spermatic cord. It provides motor fibers to the cremaster and dartos muscles and sensory fibers to the spermatic fasciae and tunica vaginalis in the testis. The nerve also causes a cutaneous feeling in the upper anterior region of the scrotum. In women, the nerve runs alongside the uterine round ligament and innervates the skin of the mons pubis and labium majus [1].

Variations in the branching pattern of the GFN are reported to be present in nearly half of the population [2-4]. The branching may manifest before the nerve enters the psoas muscle, within the muscle itself (through penetration by muscle fibers), or after the nerve exits the muscle as it traverses its course toward the inguinal ligament [5]. This variability in branching patterns highlights the complexity and intricacy of anatomical structures within the human body, emphasizing the need for a comprehensive understanding of such variations in clinical and educational contexts [6,7].

In the context of our discussion, a notable case of GFN variation in branching is presented, offering a practical example of the diverse anatomical configurations that can be encountered.

## Case presentation

**Figure 1:**
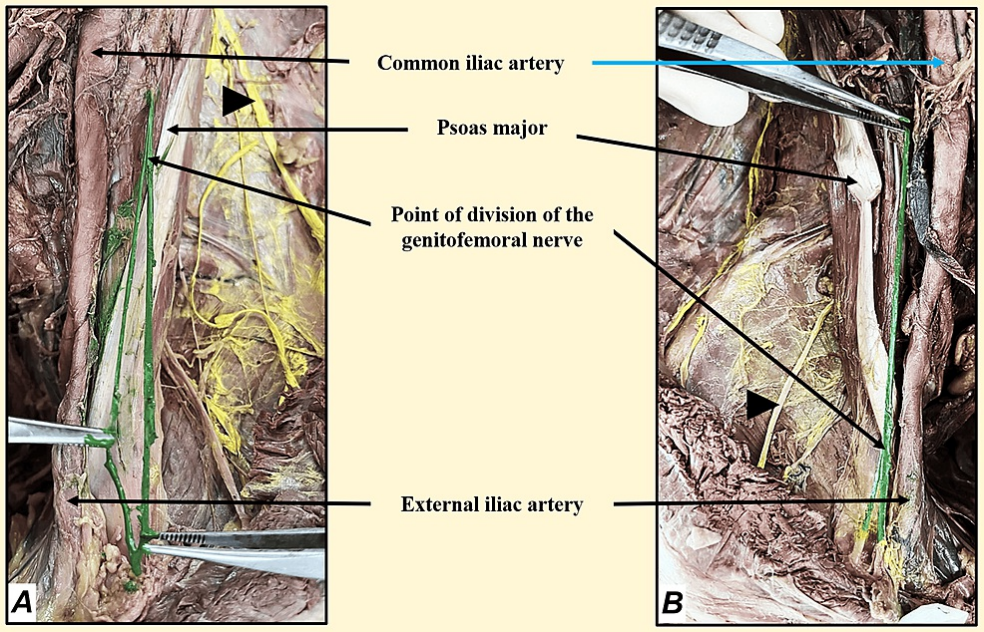
A: green-colored structure showing the higher division of the genitofemoral nerve on the left side; B: green-colored structure showing the usual division of the genitofemoral nerve on the right side; black arrowhead showing yellow coloration being done for other branches of the lumbar plexus

During an educational dissection session focusing on the posterior abdominal wall of an elderly male cadaver, an intriguing variation pertaining to the GFN was observed on the left side of the cadaver. The GFN displayed an unusual branching pattern, bifurcating just after piercing the psoas muscle and continuing its course in a manner divergent from the typical anatomical arrangement (Figure 1A). In contrast, the right-sided GFN exhibited a more conventional trajectory, dividing into its two branches just before reaching the inguinal ligament (Figure 1B).

Intrigued by this anatomical anomaly, further investigation was undertaken to trace the path of the high-branched GFN and ascertain any variations in its course. It was decided that coloring the GFN during dissection would serve as a visual aid and enhance the educational experience for the students, facilitating a deeper understanding of the significance of anatomical knowledge and variations in branching patterns.

The GFN on both sides were meticulously colored green, highlighting their course up to the points where they left the abdomen. Additionally, to draw attention to the observed variation, other discernible branches of the lumbar plexus preceding the segmental dissection of the psoas muscle were colored yellow (Figure 1), directing learners' focus towards the distinct branching patterns observed.

Furthermore, the educational session delved into the functional aspects of the GFN, particularly elucidating the mechanism of the cremasteric reflex. This comprehensive exploration encompassed the sensory and motor components of the GFN, providing students with a holistic understanding of its role in both sensory perception and motor control within the context of the abdominal and inguinal regions.

By incorporating this hands-on and visually engaging approach to anatomical education, the session aimed to impart factual knowledge and foster critical thinking and observational skills among the students. Moreover, it highlights the importance of recognizing and comprehending anatomical variations, as they hold significant implications for clinical practice, surgical interventions, and diagnostic procedures in the medical field.

The present variation was observed in a voluntarily donated cadaver for medical education and research; therefore, the institutional ethics committee waived the ethical clearance.

## Discussion

As per the literature, the GFN is the most common branch of the lumbar plexus, which presents with a variation [2,3]. This variation can be at the origin, bifurcation point, or distribution area [8]. The usual point of origin for the GFN is from spinal segments L1 and L2, but sometimes it is observed to originate from L1 to L3. It shares the usual origin with the iliohypogastric and ilioinguinal nerves [9]. The common point of origin allows these branches to cross-communicate and present with a vague abnormal sensation along the dermatomal distribution in neuropathies [10].

For GFN, different points of bifurcation have been reported as the most common form of variation, with almost 50% of the studied population presenting with some form of it [2]. The two branches may separate from the root level, either of them being entirely derived from L1 or L2 and sometimes arising from L3. The genital branch may receive some additional fibers from the T12 nerves [9]. Thereafter, the bifurcations may occur inside the psoas major muscle, giving two piercing points on the medial border of the psoas muscle across the L3-L4 level. Further, the GFN may divide upon emergence on the muscle instead of dividing lower down before the inguinal ligament. As per the literature, such post-emergence divisions can be classified into higher, middle, or lower branching, where the latter is the normal form of division [4,8,10]. The cadaveric studies of the 21^st^ century have reported variation in the origin, emergence, and branching pattern of the GFN (Table 1). Most of these studies have reported the division of the common GFN into the two branches inside the psoas muscle [2,6,11-13], while very few have observed variation of GFN post emergence [2,10], as seen in the present study. A few case reports have also observed high branching of the GFN just after emerging from the psoas muscle [5]. 

**Table 1:** Variations in the genitofemoral nerve (GFN) branching reported in the literature of the 21st century

			Branching of the GFN after piercing the muscle	
	At the root level	In the psoas muscle	High	Middle
Anloague & Huijbregts, 2009 [2]	-	26.5% (9/34)	20.60%	-
Ram et al., 2020 [4]	20%	-	-	-
Rab et al., 2001 [6]	-	42% (27/64)	-	-
Paul & Shastri, 2019 [10]	13.3% (8/60)	-	-	3.3% (2/60)
Sim & Webb, 2004 [11]	-	8.3% (5/60)	-	-
Uzmansel et al., 2006 [12]	-	42% (27/64)	-	-
Gandhi et al., 2013 [13]	-	21.7% (13/60)	-	-

Nerves from spinal segment L1 provide innervation to abdominal muscles and their derivatives. The spinal segment has three branches in the lumbar plexus that can cross-communicate and replace one another in supplying the respective myotome derivatives [9]. Therefore, variant nerve supply to these muscles has been commonly reported. For example, in the absence of either of the two branches of GFN, the ilioinguinal nerve replaces the genital branch, and the femoral branch is replaced by the lateral and anterior cutaneous nerves of the thigh. Vice versa, the area or muscle supplied by the ilioinguinal nerve and lateral femoral cutaneous nerve is reportedly replaced by the genital branch of the GFN [6,14].

Embryological explanation of these variations

During embryonic development, neurons are formed from neuroblasts derived from neural precursor cells, which are progenitor cells originating in the neural tube. Around the fourth week of embryonic development, the somite starts to develop within the paraxial mesoderm adjacent to the neural tube of the growing embryo. The somites consist of the sclerotome, myotome, and dermatome, with the myotome being the source of striated muscle formation. Each myotome is connected to a single spinal nerve at this stage, and the myotomes responsible for limb muscle formation migrate toward the limb buds. Simultaneously, the spinal nerves also migrate to their final destinations, corresponding to the locations where the muscles derived from the myotomes will eventually reside. Thus, it is evident that the muscles receiving innervations from multiple spinal segments undergo development from more than one myotome, showcasing the complexity and intricacy of embryonic morphogenesis. The exploration of these developmental processes in avian species through experimental analyses has yielded compelling evidence indicating that somites lack inherent, predetermined, or programmed migration pathways. Instead, they can remarkably respond dynamically to positional cues within their microenvironment [15,16].

As embryonic development progresses, the myotomes align along the developing limb bud's pre-axial and post-axial borders, a spatial organization crucial for the subsequent orchestration of muscle formation and positioning within the emerging limb structure. This migration of cells derived from somites is not solely governed by intrinsic factors but is significantly influenced by extrinsic signals [17]. Growth factor signals emanating from the apical ectodermal ridge of the limb bud field play a pivotal role in shaping the migratory paths of these myogenic precursors [18].

Furthermore, the migratory trajectories of these myogenic precursor cells are postulated to be modulated by acquired receptors on their surface, enabling them to interpret and respond to environmental cues, thus guiding their migration pathways. The potential involvement of transcriptional factors such as pax-3, possibly situated in the lateral domains of the somite, adds another layer of complexity to the regulatory mechanisms governing precursor migration and subsequent muscle development [17,19].

In the context of our study, a notable variation manifests in the formation of nerves, whereas no discernible variations are observed concerning the eventual anatomical structure of the muscles formed. This discrepancy suggests that the deviations observed in nerve formation within this region stem from variations in the migratory routes the myotomes undertake as they journey toward their intended destination sites. It is plausible that individual myotomes may have followed distinct pathways during their migratory phase, thereby resulting in alterations in the trajectories of the nerves they innervate [5].

Clinical importance

Genitofemoral neuropathy is a debilitating condition caused by iatrogenic nerve damage from surgical procedures like herniorrhaphy, cesarean section, appendectomy, lymph node biopsy, hysterectomy, and vasectomy. It is a severe condition that requires immediate treatment. Diagnosing genitofemoral neuralgia is challenging due to its clinical presentation overlapping with ilioinguinal neuralgia. Healthcare providers often use selective nerve blocks for diagnostic purposes and therapeutic intervention. Ilioinguinal nerve blocks are typically performed before GFN blocks due to their anatomical accessibility. Accessing the GFN is more challenging due to its retroperitoneal course and variations in branching and course [20].

Given the inherent complexities and variations associated with the GFN, understanding these anatomical variations is paramount for surgeons operating in the lower abdominal region, anesthetists administering nerve blocks, and radiological intensivists tasked with managing chronic groin pain. This knowledge informs clinical decision-making and enhances procedural precision and patient outcomes by mitigating the risks associated with nerve-related complications and ensuring effective pain management strategies [6].

## Conclusions

The GFN is known for its variability in origin, bifurcation, and branching patterns, which can affect clinical scenarios, especially in surgical procedures and lower abdominal neuropathies. The embryological basis for these variations lies in the dynamic migration of myotomes during embryonic development, which can result in diverse nerve trajectories. Understanding these variations is crucial for accurate nerve identification, diagnostic procedures, and effective management of genitofemoral neuralgia, reducing complications, and optimizing pain management strategies. Despite numerous reports of GFN variations, the literature needs to explain the accurate embryological and genetic causes. This adds to the limitations of this study while providing scope for further research.
